# An Account of an Improvement on the “Urethra Probe” of Sir Charles Bell

**Published:** 1840-01-11

**Authors:** Wm. A. Turner

**Affiliations:** Windsor, North Carolina


*An Account of an improvement on the "Urethra Probe" of Sir Charles Bell, by* WM. A. TURNER, M. D., *of Windsor, North Carolina.*

To the Editors of the Medical Examiner.

GENTLEMEN,—We were lately both edified and instructed by an examination of an improvement on the above instrument of Sir Charles Bell, made according to the directions of Dr. Turner, of North Carolina, by Mr. Warner of Philadelphia; and as we have ever been disposed to bestow praise on American inventions of merit, we must be excused for giving a short history and account of the Probe above referred to.

Doctor Turner's improvement seems admirably adapted to the treatment of prominent strictures of the urethra, and in truth we may safely say, that in point of ingenuity, convenience, and nicety of construction, it is not surpassed by any instrument of the kind ever invented in America.

It consists of a silver rod about ½ a line in diameter; its length is 10 inches; each extremity of the rod is furnished with an oval ball, one line in diameter, though perhaps the diameter of the one extremity is smaller than that of the other; 9 lines from either extremity of the probe, the rod gradually enlarges for about one inch; it then continues of uniform size for 12 lines further, and after this, declines in its transverse diameter, until it is reduced to its original size as before.

A screw, with round smooth thread, is cut on an enlargement, next the ball or extremity, and by turning the rod, this screw progresses forward through the stricture without much difficulty. The principle upon which this probe acts, is by dilating the strictured portions of the urethra, without that unpleasant feeling to the patient that is experienced in pushing a bougie into the urethra; for in the use of the latter instrument, the laxity of the proper tissue of the urethra favours the recession of the stricture, and the instrument is therefore often withdrawn under the delusive hope that the stricture is dilated, when in reality, the bougie has not come in contact with the resisting portions of the urethra at all.

Whilst in the pursuit of his professional studies in Philadelphia, a few years since, Dr. Turner (the author of the instrument now under consideration) frequently noticed the difficulty experienced by surgeons in attempting to push a bougie into the urethra. This fact induced him to form an instrument after his own suggestions: he had, therefore, a probe of the kind that we have just adverted to, made by a distinguished cutler of this city, and presented it to his friend and classmate, Dr. W. M. S. Ridley, (then of North Carolina, but since of Georgia, and one of the most intelligent, energetic, and accomplished young surgeons of which this country can boast, and destined to be at the head of the profession.) Since which time he has used the instrument himself, and his experience meets the concurrence of Dr. Ridley, as testified to by the following letter, dated, Harris, Georgia, August 26th, 1839.

"Since my last pleasure in seeing you, I have had the good fortune to relieve many cases of that annoying disease, stricture of urethra, and in each instance the cure was effected through the use of the valuable instrument which your kindness put me in possession of, during our mutual pupilage in Philadelphia. Its use has indeed surpassed my most ardent anticipations, and, in fine, I would not be deprived of it under any considerations.

"I find in the use of the probe, that if its balled extremity will pass the resisting part of the urethra, that the stricture will certainly succumb, and much more easily, too, both to operator and patient, than with any other instrument it has been my province to use. By way of suggestion, allow me to say that the instrument should be patented, and published, for it must be adopted in practice, and the honor of the invention should be accorded to the proper person. With sentiments of hearty approbation of the instrument, with my best wishes for its good success in future, I have the honour of being your friend and classmate,

Wm. M. S. Ridley, M. D."

To Dr. William A. Turner, Windsor, North Carolina.

We would here reiterate the suggestion of Dr. Ridley to Dr. Turner, to patent and publish the instrument, for why hide his light under a bushel until some transmarine aspirant get the idea, have the instrument made, and publish it to the world as his own invention?

Respectfully,

A Physician of Philadelphia.

P. S.—Upon the principle of the instrument just referred to, Dr. Turner has invented a stilet, which we have not as yet had an opportunity of using, but so soon as an occasion presents itself, we will, we hope, be allowed to record our experience in your valuable Journal.

These instruments are both to be seen at the office of the Medical Examiner, and at Ellis's Drug Store, Chesnut street.

*Philadelphia, December* 1, 1839.

